# Clinical Impact of Intracoronary Imaging in the Management of Stent Thrombosis

**DOI:** 10.3390/jcm13164667

**Published:** 2024-08-09

**Authors:** Grigoris V. Karamasis, Athanasios Katsikis, Klio Konstantinou, Gerald J. Clesham, Paul A. Kelly, Rohan Jagathesan, Francesco Prati, Christos V. Bourantas, John R. Davies, Thomas R. Keeble

**Affiliations:** 1 Cardiology Department, Essex Cardiothoracic Centre, Basildon SS16 5NL, UK; tkatsikis@gmail.com (A.K.);; 2 School of Medicine, Anglia Ruskin University, Chelmsford CM1 1SQ, UK; 3 2nd Cardiology Department, University of Athens, University Hospital Attikon, 124 62 Haidari, Greece; 4 Cardiology Department, 401 General Military Hospital of Athens, 115 25 Athens, Greece; 5 Cardiology Department, S. Giovanni Hospital, UniCamillus-Saint Camillus International University of Health Sciences, 00131 Rome, Italy; fprati@hsangiovanni.roma.it; 6 Department of Cardiology, Barts Heart Centre, Barts Health NHS Trust, West Smithfield, London EC1A 7BE, UK; cbourantas@gmail.com; 7 Centre for Cardiovascular Medicine and Devices, William Harvey Research Institute, Queen Mary University of London, Mile End Road, London E1 4NS, UK; 8 Institute of Cardiovascular Sciences, University College London, Gower Street, London WC1E 6BT, UK

**Keywords:** stent thrombosis, OCT, IVUS, intracoronary imaging

## Abstract

**Objectives:** Use of intracoronary imaging (ICI) in cases of stent thrombosis (ST) is recommended and tailored treatment appears reasonable. Nevertheless, data supporting such a strategy are lacking. The aim of this study was to evaluate the clinical impact of ICI in the management of ST. **Methods:** The unadjusted study population was consecutive patients with definite ST presenting in a single tertiary cardiac centre and undergoing percutaneous coronary intervention (PCI). The presumed major mechanism of ST was assigned according to the real-time ICI interpretation by the PCI operator. Propensity score matching was performed with regard to ICI use to form the adjusted population and Kaplan–Meier analysis was applied to compare survival free of cardiac death (CD) or target lesion revascularization (TLR). **Results:** The unadjusted population included 130 ST patients, with the majority presenting with ST-elevation myocardial infarction (STEMI) (88%) and very late ST (86%). ICI was performed in 45 patients, of whom optical coherence tomography (OCT) was performed in 30 cases. When the individual ST mechanisms were viewed as groups, there was an interaction observed between type of treatment (stent vs. non-stent) and ST mechanism, with non-stent treatment being more prevalent in cases of underexpansion, malapposition, in-stent restenosis and mechanism uncertainty. After application of matching, two groups of 30 patients were formed. ICI-guided management resulted in better survival free of CD–TLR at 2 years (93% vs. 73%, *p* = 0.037). **Conclusions:** Intracoronary imaging guidance during PCI for ST had a direct impact on management (stent vs. non-stent) and resulted in a lower event rate at mid-term follow-up when propensity matched analysis was applied.

## 1. Introduction

In the modern era of percutaneous coronary intervention, advances in stent design and antiplatelet therapy have resulted in very low rates of stent thrombosis (ST) [1,2]. Despite this achievement, ST remains the most serious post-procedural complication of stent implantation, manifesting by definition as an acute coronary syndrome (ACS) with frequently devastating consequences [3,4]. Compared to non-ST related ACS, ST has a different, more complex and less well understood pathophysiology, but at the moment there is no evidence that the interventional management of patients with ST should differ from that of patients with acute thrombosis of a de novo lesion. Intracoronary imaging (ICI) can delineate the underlying mechanisms of ST and current international guidelines and consensus documents recommend its use in the setting of ST [5,6,7]. A tailored approach based on the findings of ICI (i.e., balloon dilatation in case of underexpansion and malapposition or stent implantation in cases of neoatherosclerosis, stent edge dissection and residual plaque/disease) seems reasonable and is supported by contemporary literature [7,8,9,10]. In clinical practice, redo stenting for ST is used in 30–60% of cases [3,11,12,13]. Nevertheless, clinical data supporting the use of ICI in this clinical scenario are limited. The aim of this study was to evaluate the clinical impact of ICI in the management of ST.

## 2. Materials and Methods

### 2.1. Study Design

The initial patients’ pool for the study was the Essex Stent Thrombosis Investigation Registry (ESTHIR). The registry has been described in detail before [13]. In brief it involved all patients with definite ST [14] managed at a tertiary cardiac centre (Essex Cardiothoracic Centre, Basildon, UK) with an initial strategy of urgent angiography from November 2015 to June 2018. Final management mode in this registry was classified as either new stent implantation or non-stent treatment. The latter was sub-classified in a hierarchical manner as medical management (combination of antiplatelet and/or antithrombotic therapy), thrombo-aspiration, plain old balloon angioplasty (POBA) and drug-coated balloon angioplasty (DCB).

Follow-up was performed by means of hospital records review and General Register Office data to document all-cause death (ACD), cardiovascular death (CD), and target lesion revascularization (TLR). The primary endpoint of the study was defined as survival free of CD or TLR [14]. The cause of death documented in the study was the primary cause of death as reported in the national General Register Office that holds records of all deaths in England and Wales. CD included deaths that resulted from an acute myocardial infarction, sudden cardiac death, death due to heart failure, death due to stroke, death due to cardiovascular (CV) procedures, death due to CV haemorrhage, and death due to other CV causes [14].

**Figure 1:**
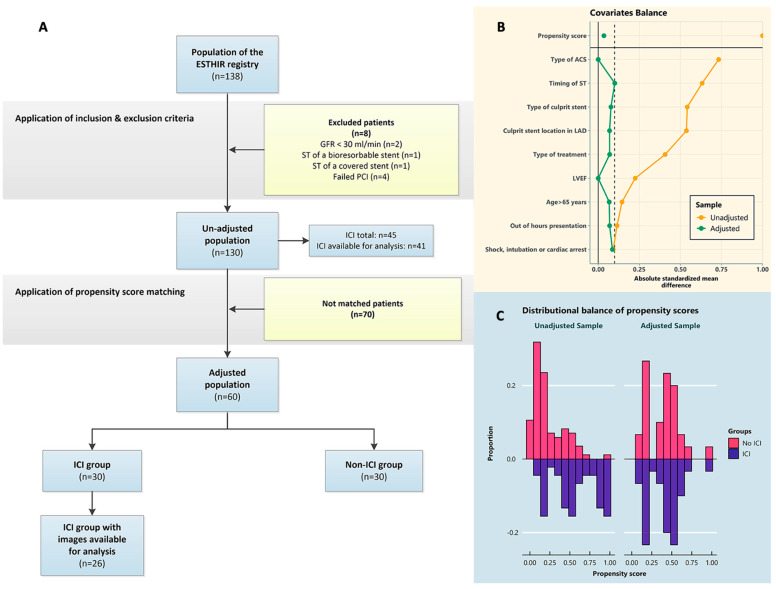
Study flowchart and overall performance of the matching process. (**A**) Study flowchart. (**B**) Covariates balance. (**C**) Distributional balance of propensity scores. ACS = acute coronary syndrome, GFR = glomerular filtration rate, ICI = intracoronary imaging, LAD = left anterior descending, LVEF = left ventricular ejection fraction, PCI = percutaneous coronary intervention, ST = stent thrombosis.

The unadjusted population of this study was formed according to the following inclusion criteria: ST of either a drug eluting stent (DES) or a bare metal stent (BMS), management with either a DES or non-stent treatment, and successful intervention, defined as residual stenosis < 10% with thrombolysis in myocardial infarction (TIMI) flow ≥ 2 in the culprit vessel and no significant side branch occlusion or flow-limiting dissection after intervention. The only exclusion criterion was estimated glomerular filtration rate (eGFR) < 30 mL/min. Subsequently, the adjusted population was extracted after matching with regard to the use of intra-coronary imaging, as intention to treat. The flowchart of the study is presented in Figure 1A. All patients provided written informed consent for the index procedure. The study was conducted according to the principles set by the Declaration of Helsinki and was eligible for waiver of ethical committee review due to its observational nature.

### 2.2. Intravascular Imaging and Classification of ST Mechanism

ICI was at the discretion of the interventional cardiologist performing the index percutaneous coronary intervention (PCI), with no pre-specified criteria regarding the type of ICI, the evaluation of ST mechanism, or further procedural planning and optimization. Patients who underwent ICI had either optical coherence tomography (OCT) using the Dragonfly imaging catheter (Abbott Vascular/St. Jude Medical, St. Paul, MN, USA) or intravascular ultrasound (IVUS) imaging using the Eagle Eye catheter (Philips/Volcano Therapeutics, Rancho Cordova, CA, USA).

Classification of ST mechanism was performed according to the real-time interpretation of the ICI findings, as described by the PCI operator in the procedural report. The presumed major mechanism was assigned to one of the following categories based on previous literature: underexpansion, malapposition, neoatherosclerosis, in-stent restenosis, edge-related disease progression (ER-DP), and edge dissection [15,16]. If none of these was documented, the label uncertain mechanism was assigned. Furthermore, the ST mechanisms were grouped in 2 categories for management-based analysis, depending on whether it would be reasonable according to contemporary literature to treat the ST without placing a new stent [7,8,9,10]. The type A category included neo atherosclerosis, ER-DP and edge dissection, and type B included underexpansion, malapposition, in-stent restenosis and uncertain. Finally, regarding timing of ST, a binary classification of early and delayed ST was used, with the first category including acute and sub-acute and the second late and very late ST [14].

### 2.3. Statistical Analysis

The normality assumption for continuous variables was evaluated by the Shapiro–Wilk test. Continuous and normally distributed variables are presented as mean ± standard deviation while continuous variables with asymmetric distribution are presented as median with range values. Categorical data are presented as counts and percentages. Between groups differences were examined by the Pearson Chi-square/Fisher’s exact test and the Mood’s median test for independent samples as appropriate. Propensity score matching analysis was performed to adjust in regard to ICI use. The propensity score was obtained by logistic regression analysis with ICI as the dependent variable and the following parameters as independent variables: age > 65 years, type of ST (early vs. delayed), type of presentation [ST-elevation myocardial infarction (STEMI) vs. non ST-elevation myocardial infarction (NSTEMI)], type of culprit stent (BMS or DES), location of culprit stent (left anterior descending artery (LAD) vs. non-LAD), type of treatment used (stent vs. non-stent), left ventricular ejection fraction (LVEF), out of hours (OOH) presentation and the composite of presentation with cardiac arrest, cardiogenic shock, or mechanical ventilation during management. Nearest neighbour matching without replacement was implemented by applying a “greedy” algorithm on the logit of the propensity score within a calliper equal to 0.13 times the pooled standard deviation of the logit. The effectiveness of matching to alleviate covariate imbalance was evaluated by the standardised difference “d” of the covariates between treatment groups, with a d ≤ 10% being considered as an indication of negligible differences in the mean or prevalence of covariates between groups. In the adjusted population, Kaplan–Meier analysis was used to evaluate event-free survival between ICI-based groups. Matching was performed using the Matching package and covariate balance was assessed using the Cobalt package of R software version 3.5.1 while all statistical analyses were carried out with PASW version 22 (SPSS, Inc., Chicago, IL, USA). All tests were 2-tailed, and a *p* value < 0.05 was considered statistically significant.

## 3. Results

A total of 130 patients presenting with ST between November 2015 and June 2018 formed the initial population of the study. The vast majority of them presented with STEMI (88%) and very late ST (VLST) (86%). ICI was used in 45 patients (35%), of which OCT was used in 29 cases, IVUS in 15 and one case used both modalities. Baseline and peri-procedural characteristics of the unadjusted and adjusted (after matching) samples are presented in Table 1 and Table 2. In the overall/unadjusted population there were differences between the ICI and non-ICI groups, most notably in the type of acute coronary syndrome (ACS) (i.e., STEMI vs. NSTEMI), type and location of thrombosed stent, and type of ST.

After propensity matching (Figure 1A), two groups of 30 patients were formed. In these two groups, all baseline characteristics that were used for matching demonstrated standardized mean differences (SMDs) well below 10%, with non-significant *p* values in pairwise comparisons. This was also the case for the only non-baseline characteristic that was used for matching, i.e., type of treatment used (7%, *p* > 0.9). None of the baseline characteristics that were not used for matching demonstrated statistically significant differences in the matched population. However, gender, smoking, dyslipidaemia, hypertension, history of myocardial infarction (MI), anatomical extent of coronary artery disease (CAD) and mechanical ventilation demonstrated SMDs > 25% between ICI and non-ICI group in the adjusted population. The overall performance of the matching process is demonstrated graphically in Figure 1B,C.

### 3.1. Intracoronary Imaging and Its Effect on PCI Procedure

Two imaging studies were judged uninterpretable, and in four other cases ICI was attempted but eventually abandoned due to inability to pass the imaging probe through the lesion. Hence, 39 ST patients were available for analysis, 22 of whom were treated with an additional stent.

**Figure 2:**
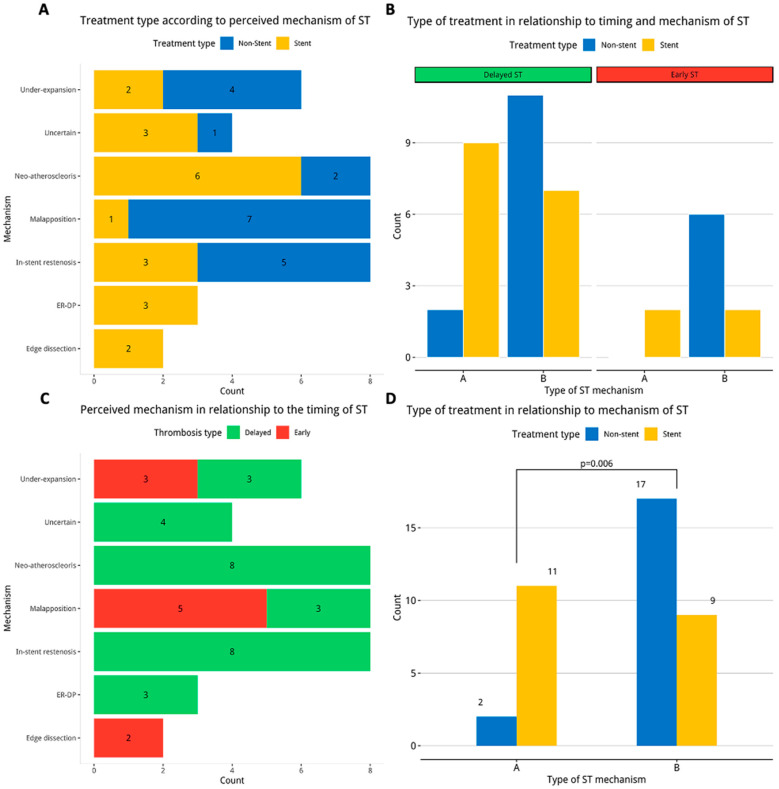
Perceived stent thrombosis (ST) mechanism, timing, and type of treatment. (**A**) Treatment type according to perceived ST mechanism. (**B**) Treatment type in relation to timing and mechanism of ST. (**C**) Perceived mechanism in relationship to timing of ST. (**D**) Type of treatment in relation to mechanism of ST. Type A mechanisms = neoatherosclerosis, ER-DP and edge dissection. Type B mechanisms = und–expansion, malapposition, in-stent restenosis and uncertain. ER-DP = edge related disease progression, ST = stent thrombosis.

The classification of the ST mechanism based on the real-time interpretation of the ICI findings is demonstrated graphically in Figure 2A. The leading causes of ST were neoatherosclerosis (20%), malapposition (20%), in-stent restenosis (20%), and underexpansion (15%), while the mechanism was uncertain in 10%. Significant differences in the management strategy (stent vs. non-stent) of different mechanisms of ST were observed (*p* = 0.006, Figure 2D). Type A mechanisms were more likely to be treated without stent implantation, while type B mechanisms were often treated with additional stents. When the individual ST mechanisms observed were considered in association with the timing of ST, neoatherosclerosis, in-stent restenosis, ER–DP, and uncertain mechanisms were observed exclusively in delayed ST, while edge dissection was observed only in early ST (Figure 2C).

Table 2 presents the comparison of the peri-procedural characteristics between the ICI and non-ICI groups. In the unadjusted population, significant differences in the mode of treatment were present between ICI and non-ICI groups. In the adjusted population, patients who had ICI-guided PCI had significantly more frequent thrombus aspiration as an initial treatment to restore flow, but similar rates of thrombus aspiration as a destination therapy. Use of IIb/IIIa was also similar between the two groups. The specific mode of treatment, rate of non-culprit vessel PCI during ST management, and type of antiplatelet therapy post-intervention did not demonstrate significant differences between the two groups.

**Figure 3:**
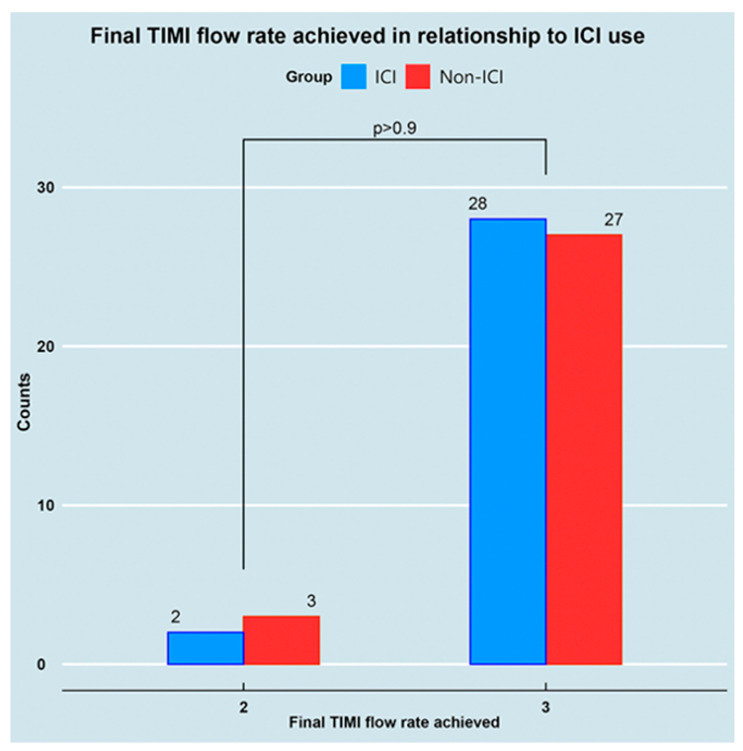
Final TIMI flow in relation to intracoronary imaging (ICI) use. TIMI = thrombolysis in myocardial infarction.

Table 2 summarizes the immediate angiographic results obtained with ICI guided management. As shown in Figure 3, the ICI group achieved similar rates of TIMI 3 flow post PCI (93% vs. 90%, *p* > 0.9). There were no differences in the number of implanted stents or the total length of stents used.

### 3.2. Effect of Intracoronary Imaging on Clinical Outcomes

**Figure 4:**
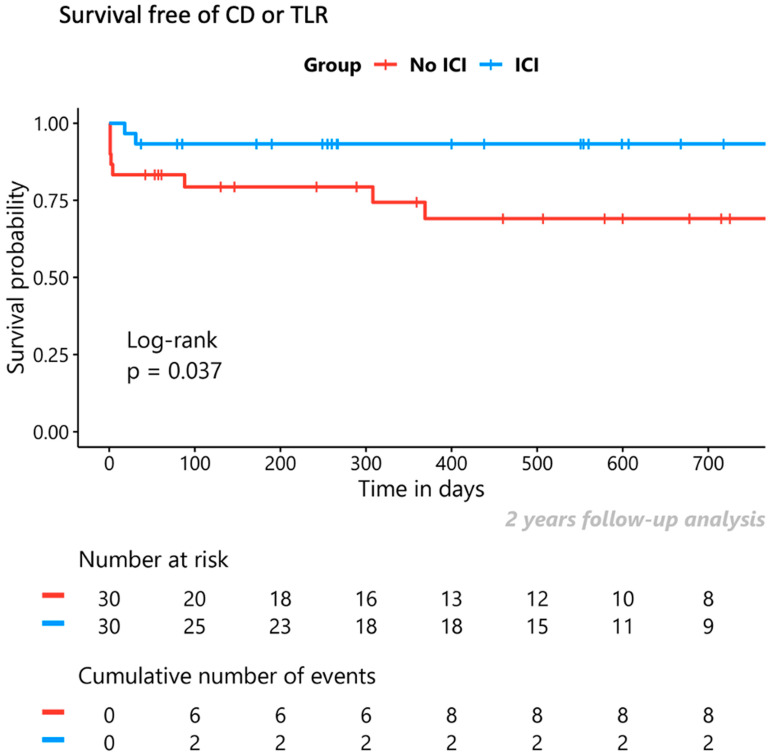
Impact of ICI on clinical outcomes. Kaplan–Meier survival analysis for the combined endpoint of cardiac death (CD) or target lesion revascularisation (TLR). ICI = intracoronary imaging.

At 2 years of follow-up, 20 ACDs, 14 CDs, and 7 TLRs (6 of them in the non-ICI group) were observed in the unadjusted sample. In the adjusted sample, 16 of these events were included, with their breakdown being: 14 ACDs, 8 CDs, and 2 TLRs, both of which were performed in patients in the non-ICI group, at 5 and 88 days after initial PCI. In the adjusted sample, the ICI group demonstrated similar rates of survival free of ACD (80% vs. 73%, *p* = 0.4) and CD (93% vs. 80%, *p* = 0.1) compared to the non-ICI group at 2 years of follow-up. However, with regard to the combined primary endpoint of CD or TLR, ICI guided management resulted in better event-free survival compared to non-ICI-guided management for the same period (93% vs. 73%, *p* = 0.037). Figure 4 demonstrates the Kaplan–Meier survival analysis according to the use of ICI for the combined endpoint of CD or TLR.

## 4. Discussion

The present study demonstrated that, in a contemporary management environment, patients with ST receiving ICI-guided treatment fared better, regardless of the actual method of management that was applied (stent vs. non-stent treatment). To the best of our knowledge, this is the first study that has evaluated outcomes following ICI-guided PCI of patients, with ST showing a clinical benefit in ICI in this setting. Although this benefit was observed for a combined clinical endpoint, the ability to demonstrate a significant difference in event-free survival in such a small, pilot trial has high clinical translation potential in a field where management data remain limited.

ST remains a therapeutic challenge and there are no current international guidelines to support specific treatment and differentiate management from native vessel thrombosis, despite the distinct pathologies [17]. ST is a rare event, making the conduction of randomised controlled trials evaluating the efficacy of different treatment strategies difficult. Three cohort studies have shed light on correlates of ST using OCT [12,15,18]. The observed underlying findings differed, depending on the timing of ST: in early ST, malapposition, underexpansion, and edge dissections were the predominant abnormalities, while in very late ST, malapposition, neoatherosclerosis, uncovered struts, and underexpansion were frequently observed. Similarly to these findings, the vast majority of ST cases in our study involved very late ST and patients presenting with STEMI. Furthermore, the perceived mechanism in relation of timing of ST reflected the above findings.

The improved clinical outcomes in the ICI-guided group in this study is a novel and interesting finding. IVUS-guided PCI has shown improved clinical outcomes in all comers and complex lesions in large randomised controlled trials [19,20]. OCT guidance has shown improved clinical outcomes for complex bifurcation PCI and, although it did not lead to a significant reduction in the composite endpoint of target vessel failure in a wider PCI population, it reduced the incidence of ST [21,22]. Nevertheless, patients undergoing PCI for ST cases have not been included in the above trials. A potential explanation of the improved outcomes in our study is the tailoring of ST management based on what was considered to be the main underlying mechanism. For example, in cases where underexpansion or malapposition were identified as the major findings, a further stent would be unnecessary, potentially increasing the risk of future stent failure. On the other hand, in cases of neoatherosclerosis or stent edge-related disease, balloon angioplasty could be insufficient in preventing further events. The presumed mechanisms of ST affected treatment planning, with patients with neoatherosclerosis, edge-related disease progression and edge dissection receiving more stents than patients with underexpansion or malapposition. Characteristically, all five cases of edge-related disease progression and edge dissection were treated with a new stent, while seven out of the eight cases where malapposition was considered the main finding were treated with balloon angioplasty. Nevertheless, it should be emphasised that there is still a long way to go in order to establish links between ICI findings, treatment, and improvement of outcomes in ST management, especially taking into account that there was no pre-specified treatment protocol or optimisation criteria for the current study. Furthermore, beyond the delineation of the stent failure mechanism, ICI was employed for procedural planning and optimisation (i.e., stent sizing), which might have influenced the procedural and clinical outcomes.

The underlying mechanism of ST thrombosis was assigned based on the real-time ICI interpretation by the PCI operator, as the purpose of the study was not to describe imaging findings of ST but to assess the feasibility and impact of ICI in a real-life scenario. PCI operators intended to use ICI in 45 cases, although in four cases the imaging probe could not cross the lesion and in two cases the images acquired were of poor quality and uninterpretable. It is not a surprise that there were cases where the imaging catheter could not be passed through the old stent, due to tortuosity, calcification, or tight stenosis. Furthermore, excessive thrombus or poor blood clearance can make the images uninterpretable, and this has been described in the previous studies using ICI in the context of ST [12,15]. Nevertheless, in the vast majority of the cases, ICI was feasible, and provided comprehensive images that allowed treatment planning. In the major OCT studies evaluating ICI findings in patients with ST, one or several underlaying mechanisms could be identified in most patients (>90%) [7]. Nevertheless, all of these studies have used offline, core lab-based, fully quantitative analysis. Such an analysis is very difficult to perform in real-time in an acute setting that calls for rapid decision making and application of the selected management. Our study provides additional data to support the consensus-based recommendations for ICI use in the management of ST in a very practical fashion, showing that the benefit of direct integration of the imaging information in the decision making process outweighs the limitations of image analysis in the cath lab. Regarding agreement of online and core lab analysis, in the PESTO (Morphological Parameters Explaining Stent Thrombosis assessed by OCT) registry, the interpretations of the operating physicians agreed with those of experts in 70% of cases [12]. Nevertheless, in that study, the analysed OCT runs were acquired during a deferred procedure in 69% of the patients, with a median delay of 4 days between initial presentation and OCT imaging. OCT images acquired during the deferred procedure should be more easily interpretable, as time delay and antithrombotic treatments would have reduced the amount of thrombus, facilitating image interpretation.

In our study, both OCT and IVUS were used for the management of ST, as there was no pre-specified protocol and modality selection was based on operator’s preference and expertise. Nevertheless, at the moment, OCT is considered the preferred technique to study ST [7]. OCT is better than IVUS in distinguishing thrombus from other tissue components. Furthermore, it has superior capacity in detecting neoatherosclerosis [7,9,23,24].

## 5. Limitations

The major limitation of the present study is its sample size which limits the statistical power of the conclusions reached, predominantly by not allowing all of the patients undergoing ICI in the registry to be matched. Nevertheless, the sample size is comparable to other studies in the field and the rarity of ST renders the design of prospective randomised controlled trials very challenging. Furthermore, this is a single centre, propensity score matched study. A multicentre, adequately powered randomised controlled trial would be necessary to reach definite conclusions. Regarding the propensity score matching, some residual imbalances in the unmatched characteristics could be observed in the adjusted population, despite the absence of statistically significant differences in pairwise comparisons. Residual imbalances and unmeasured confounders are well described limitations applicable to all propensity score matching studies. All decisions were left to operators’ discretion and there were no pre-specified criteria for ICI use, interpretation of its findings or ST optimal management. Detailing a pre-specified process of ICI interpretation and decision-making could have provided more insight into the clinical implications. Furthermore, no core lab analysis or adjudication of ICI findings was performed. Nevertheless, the intention of the study was not to report causes of ST, but to assess the impact of ICI in guiding revascularisation in real-time. Both IVUS and OCT cases were included in the study, as this methodology has been used before in the literature about stent thrombosis [23,25]. Nevertheless, the two modalities have different sensitivities and performances to analyse ST underlying findings. IVUS can clearly visualise tissue proliferation within the stent (ISR), but its ability to discriminate between the two main pathological substrates of ISR, neoatherosclerosis and neointimal hyperplasia [26], is limited (especially if not a high-definition device, as in this study) [7,9,23,24]. Therefore, following the previous literature about IVUS in ST cases, we included ISR as a separate ICI finding [16]. Yet, the use of either OCT or IVUS reflects real-life practice when delineating stent failure and the choice is based on availability and PCI operator’s expertise or preference. Finally, the applied treatment strategies could have been influenced by patients and procedural characteristics not captured in the current data set (i.e., frailty, presence of calcium, or tortuosity).

## 6. Conclusions

This study describes the clinical impact of ICI-guided management of ST. It showed that ICI-guided management with real-time interpretation of findings was feasible and could be the basis for a tailored treatment approach. Furthermore, in the propensity matched population, ICI resulted in better mid-term clinical outcomes. Although the results of the study are important, they should be interpreted with caution due the non-randomised study design and the relatively small sample size. Future, larger studies should explore further the role of ICI in the management of ST.

### Impact on Daily Practice

Use of intracoronary imaging (ICI) in cases of stent thrombosis (ST) is recommended by international guidelines and a tailored treatment appears reasonable. In this study, ICI guidance during PCI for ST had a direct impact on invasive management (stent vs. non-stent treatment) and resulted in a lower adverse event rate at mid-term follow-up in the propensity matched population.

## Figures and Tables

**Table 1:** Baseline characteristics.

Baseline Characteristics			Unadjusted Population				Matched/Adjusted Sample			
			No ICI	ICI			No ICI	ICI		
			(n = 85)	(n = 45)	*p*-Value	SMD (%)	(n = 30)	(n = 30)	*p*-Value	SMD (%)
**Demographics**										
Age (years)			65 (56,73)	63 (53,71)	0.2	27	62 (55,72)	64 (52,71)	0.8	3
Male gender (n,%)			72 (85%)	38 (84%)	>0.9	1	28 (93%)	25 (83%)	0.4	27
**Cardiovascular** **risk factors**										
Active smoking (n,%)			35 (41%)	8 (18%)	0.012	61	13 (43%)	6 (20%)	0.052	67
Dyslipidaemia (n,%)			61 (72%)	22 (49%)	0.017	45	22 (73%)	15 (50%)	0.11	46
Hypertension (n,%)			65 (76%)	21 (47%)	0.001	59	22 (73%)	16 (53%)	0.2	40
Diabetes (n,%)			20 (24%)	10 (22%)	>0.9	3	6 (20%)	5 (17%)	>0.9	8
Family history (n,%)			20 (24%)	12 (27%)	0.9	7	5 (17%)	7 (23%)	0.7	15
BMI			28.1 (25.5,29.4)	28.6 (25.1,31.1)	0.3	17	27.6 (25.6,28.6)	28.6 (25.8,31.5)	0.13	21
Chronic kidney disease ^#^ (n,%)			15 (18%)	5 (11%)	0.5	21	5 (17%)	3 (10%)	0.7	21
**CAD status** (n,%)										
Previous MI			74 (87%)	33 (73%)	0.087	31	28 (93%)	23 (77%)	0.15	37
Previous CABG			9 (11%)	5 (11%)	>0.9	2	3 (10%)	1 (3.3%)	0.6	21
Number of coronary arteries diseased (n,%)	1		55 (65%)	34 (76%)	0.3	33	19 (63%)	23 (77%)	0.2	43
	2		20 (24%)	9 (20%)			8 (27%)	7 (23%)		
	3		10 (12%)	2 (4.4%)			3 (10%)	0 (0%)		
**LVEF (%)**			44 (41,55)	45 (35,55)	>0.9	20	43 (40,49)	45 (40,45)	0.7	0
**Presentation mode:** **Clinical** (n,%)										
Cardiogenic shock			8 (9.4%)	4 (8.9%)	>0.9	2	4 (13%)	3 (10%)	>0.9	12
Mechanical ventilation			7 (8.2%)	2 (4.4%)	0.5	18	4 (13%)	1 (3.3%)	0.4	48
Post-cardiac arrest			10 (12%)	7 (16%)	0.7	10	5 (17%)	5 (17%)	>0.9	0
STEMI			82 (96%)	32 (71%)	<0.001	55	27 (90%)	27 (90%)	>0.9	0
NSTEMI/UA			3 (3.5%)	13 (29%)			3 (10%)	3 (10%)		
OOH presentation			33 (39%)	15 (33%)	0.7	12	12 (40%)	11 (37%)	>0.9	7
Discontinuation of APT			25 (29%)	9 (20%)	0.3	23	9 (30%)	8 (27%)	>0.9	8
**Presentation mode:** **Stent related**										
Type of ST (n,%)		Acute	0 (0%)	8 (18%)	<0.001 (*)	48 (*)	0 (0%)	3 (10%)	>0.9 (*)	8 (*)
		Sub-acute	2 (2%)	2 (4%)			1 (3%)	1 (3%)		
		Late	3 (3%)	3 (7%)			3 (10%)	1 (3%)		
		Very late	80 (94%)	32 (71%)			26 (87%)	25 (83%)		
Initial TIMI flow (n,%)	>0		24 (28%)	18 (40%)	0.2	24	11 (37%)	12 (40%)	>0.9	7
	0		61 (72%)	27 (60%)			19 (63%)	18 (60%)		
Culprit vessel: LAD vs. non-LAD (n,%)			29 (34%)	27 (60%)	0.008	52	17 (57%)	18 (60%)	>0.9	7
Culprit stent type (n,%)		DES overall	52 (61%)	38 (84%)	0.011 (**)	63 (**)	25 (83%)	24 (80%)	>0.9 (**)	9 (**)
		BMS	33 (39%)	7 (65%)			5 (17%)	6 (20%)		
		DES 1st gen.	13 (15%)	6 (13%)			6 (20%)	6 (20%)		
		DES 2nd gen.	39 (46%)	32 (71%)			19 (63%)	18 (60%)		

(^#^) Chronic kidney disease was defined as an eGFR ≥ 30 mL/min and <60 mL/min. Patients with eGFR < 30 mL/min were excluded from the study. (*) For early vs. delayed), (**) For DES vs. BMS. Abbreviations: APT = antiplatelet therapy, BMI = body mass index, BMS = bare metal stent, CABG = coronary artery bypass grafting, CAD = coronary artery disease, DES = drug eluting stent, gen = generation, ICI = intracoronary imaging, LAD = left anterior descending, LVEF = left ventricular ejection fraction, MI = myocardial infarction, NSTEMI = non ST-elevation myocardial infarction, OOH = out of hours, SMD = standardized mean differences, ST = stent thrombosis, STEMI = ST-elevation myocardial infarction, TIMI = thrombolysis in myocardial infarction, UA = unstable angina.

**Table 2:** Peri-procedural characteristics.

Peri-Procedural Characteristics	Unadjusted Population			Propensity Matched/Adjusted Sample		
	No ICI	ICI		No ICI	ICI	
	(n = 85)	(n = 45)	*p*-Value	(n = 30)	(n = 30)	*p*-Value
**Culprit stent localization**						
Graft	2 (2%)	2 (4%)	0.046	0 (0%)	0 (0%)	0.8
LAD	27 (32%)	26 (58%)		15 (50%)	17 (57%)	
Circumflex	11 (13%)	6 (13%)		3 (10%)	4 (13%)	
RCA	42 (49%)	10 (22%)		10 (33%)	8 (27%)	
Diagonal	2 (2%)	1 (2%)		2 (7%)	1 (3%)	
Intermediate	1 (1%)	0 (0%)		0 (0%)	0 (0%)	
**Thromboaspiration**	43 (51%)	31 (69%)	0.069	13 (43%)	23 (77%)	0.018
**IIb/IIIa use**	45 (53%)	29 (64%)	0.3	17 (57%)	19 (63%)	0.8
**End TIMI flow**						
End-TIMI flow = 2	9 (11%)	4 (8.9%)	>0.9	3 (10%)	2 (6.7%)	>0.9
End-TIMI flow = 3	76 (89%)	41 (91%)		27 (90%)	28 (93%)	
**>1 stent per culprit vessel**	18 (21%)	4 (9%)	0.07	4 (13%)	2 (7%)	0.7
Multi-vessel PCI **	4 (4.7%)	5 (11%)	0.3	3 (10%)	3 (10%)	>0.9
**Type of treatment**						
Thromboaspiration	2 (2%)	0 (0%)	0.045 (*)	1 (3%)	0 (0%)	>0.9 (*)
DCB	13 (15%)	3 (7%)		7 (23%)	2 (7%)	
DES	60 (71%)	23 (51%)		17 (57%)	18 (60%)	
In-hospital CABG	0 (0%)	1 (2%)		0 (0%)	0 (0%)	
POBA	10 (12%)	18 (40%)		5 (17%)	10 (33%)	
**Post- intervention APT**						
ASA	1 (1.2%)	0 (0%)	0.4	0 (0%)	0 (0%)	0.2
ASA + Clopidogrel	19 (22%)	9 (20%)		9 (30%)	9 (30%)	
ASA + Prasugrel	3 (3.5%)	4 (8.9%)		0 (0%)	3 (10%)	
ASA + Ticagrelor	62 (73%)	31 (69%)		21 (70%)	17 (57%)	
Unknown	0 (0%)	1 (2.2%)		0 (0%)	1 (3.3%)	

* For stent vs. non-stent treatment. ** Regardless of exact type of treatment (i.e., balloon angioplasty or stenting). Abbreviations: APT = antiplatelet therapy, ASA = aspirin, CABG = coronary artery bypass grafting, DCB = drug coated balloon, DES = drug eluting stent, ICI = intracoronary imaging, LAD = left anterior descending, PCI = percutaneous coronary intervention, POBA = plain old balloon angioplasty, RCA = right coronary artery, TIMI = thrombolysis in myocardial infarction.

## Data Availability

Data are available on demand.
